# Bimetallic gold and palladium nanoparticles supported on copper oxide nanorods for enhanced H_2_O_2_ catalytic reduction and sensing†

**DOI:** 10.1039/d1ra05247k

**Published:** 2021-08-26

**Authors:** Simbongile Sicwetsha, Omotayo Adeniyi, Philani Mashazi

**Affiliations:** Chemistry Department, Rhodes University PO Box 94 Makhanda 6140 South Africa p.mashazi@ru.ac.za; Institute for Nanotechnology Innovation, Rhodes University PO Box 94 Makhanda 6140 South Africa

## Abstract

The emergence of nanoscience and nanotechnology has revitalised research interest in using copper and its derived nanostructures to find exciting and novel applications. In this work, mono- and bimetallic gold and palladium nanoparticles supported on copper oxide nanorods (CuONRs) were prepared and their catalytic performance towards the reduction of H_2_O_2_ to form reactive oxygen radical species (ROS) was evaluated. The characterisation using microscopy and spectroscopic techniques confirms the successful synthesis of CuONRs, CuONRs@Au_6_NPs, CuONRs@Pd_6_NPs and CuONRs@Au_3_Pd_3_NPs. The efficient generation of ROS was confirmed using UV-vis spectroscopy and 1,3-diphenylisobenzofuran (DPBF) as a radical scavenger. The CuONRs possess excellent catalytic reduction activity for H_2_O_2_ by generating ROS. However, CuONRs also have lattice oxygens which do not participate in the catalytic reduction step. The lattice oxygens however allowed for the adsorption of gold and palladium nanoparticles (Au_6_NPs, Pd_6_NPs and Au_3_Pd_3_NPs) and thus enhanced catalytic reduction of H_2_O_2_ to produce ROS. The produced ROS was subsequently involved in the catalytic oxidation of a chromogenic substrate (TMB), resulting in blue coloured diimine (TMBDI) complex which was monitored using UV-vis and could also be observed using the naked eye. The catalyst dependence on pH, temperature, and H_2_O_2_ concentration towards efficient ROS generation was investigated. The gold and palladium-supported CuONRs nanocatalysts were evaluated for their potential applications in the fabrication of colorimetric biosensors to detect glucose oxidation by glucose oxidase (GOx). Glucose was used as a model analyte. The enzymatic reaction between GOx and β-d-glucose produces H_2_O_2_ as a by-product, which is then catalytically converted to ROS by the nanoparticles.

## Abstract 2

Mono- and bimetallic gold and palladium nanoparticles supported on copper oxide nanorods were prepared. Their catalytic performance towards the catalytic reduction of H_2_O_2_ to produce reactive oxygen radical species was evaluated.

## Graphical abstract

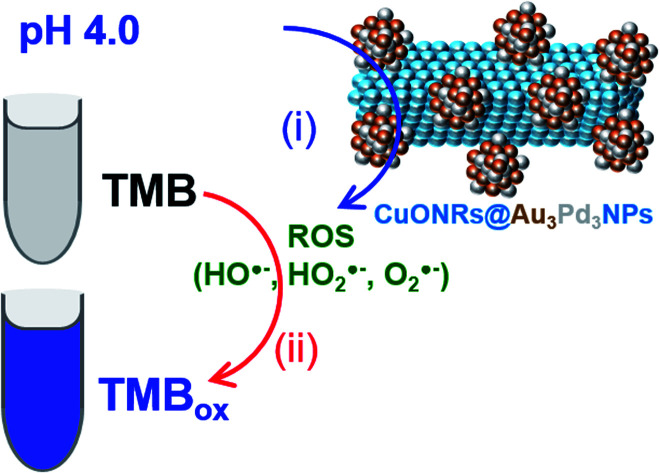

## Introduction

Bimetallic noble metal nanoparticles have received extensive research attention due to their intriguing synergistic properties and various applications in nanoscience and nanotechnology. They have been found to possess wide applications and of interest to this work is the applications as nanocatalysts in heterogeneous catalysis.^1,2^ In addition to their unique catalytic properties, nanocatalysts have found uses in various fields, including electronics, electrocatalysis, photocatalysis, and over the past two decades in nanozymology and biosensing. Integrating noble metal nanoparticles and inexpensive transition metal oxide nanoparticles into a single nanostructure produces cheaper and highly stable nanocatalysts with synergistic properties.^3^ The morphology, internal structure, and catalytic efficiency of metal nanoparticles supported onto metal oxide nanoparticles (NM-MOs) can be achieved by altering the molar ratios of the precursors and the method used for preparation.^4–6^ The composition, structure, particle size and other auxiliary variables dictate the applications of nanostructured catalysts.^7–9^ Noble metal nanoparticles supported onto metal oxide nanoparticles exhibit better catalytic performance than their monometallic analogues.^10–12^ The improved catalytic performance can be ascribed to the synergistic effect induced by altering the electronic structure between the support material (metal oxide) and interfacial noble metals.^10,13^ The size of the noble metal nanoparticles and the nature of the metal oxide support was found to be essential in determining the catalytic performance.^14,15^ One dimensional (1D) nanomaterials such as nanowires, nanotubes and nanorods have attracted significant research attention as supports for noble metal nanocatalysts. They have high surface-to-volume ratio and sometimes offer fast electron transfer process in their synergistic applications.^16^ Therefore, small size noble metal nanoparticles such as gold nanoparticles supported on 1D metal oxide nanoparticles would be efficient as nanocatalysts.^11,15^

The evolution of nanozymology has afforded scientists an insight into the enzyme-like properties of heterogeneous catalysts and their ability to mimic natural enzymes, thus their applications as artificial enzymes.^17,18^ Natural enzymes possess exceptional catalytic efficiency, substrate specificity, and high selectivity, resulting in their applications in biosensor developments.^19^ However, natural enzymes have some limitations that impede their wide industrial applications. These include low stability in harsh conditions, relatively high cost for preparation, purification and storage, narrow pH windows for optimal operation, and short shelf life.^17,19^ Artificial enzymes show superiority to natural enzymes due to low cost, high temperature and long-time storage stability.^19^ Therefore, artificial enzymes have been developed as potential substitutes for natural enzymes. Iron oxide nanoparticles were the first inorganic nanomaterials to display enzyme-like (peroxidase) activity.^20,21^ Various noble metals such as gold nanoparticles (AuNPs), platinum nanoparticles (PtNPs) and palladium nanoparticles (PdNPs), and metal oxide nanoparticles such as CeO_2_, LaNiO_3_ and V_2_O_5_ (ref. 22) have also demonstrated the various enzyme-like activity.^23^ Therefore, there is a continued need to find nanomaterials with enhanced enzyme-like activity and investigate their mechanism of action.

In this work, we report the synthesis of CuONRs as support for noble metal nanocatalyst (Au_6_NPs, Pd_6_NPs and bimetallic Au_3_Pd_3_NPs) and evaluated their peroxidase-like activity. The CuONRs possess excellent catalytic reduction properties towards H_2_O_2_ generating ROS. However, CuONRs also has lattice oxygen which do not participate in the catalytic reduction. The lattice oxygens are suitable for the adsorption and deposition noble metal nanocatalysts of gold and palladium such as Au_6_NPs, Pd_6_NPs and Au_3_Pd_3_NPs. Hence enhancing the catalytic reduction of CuONRs towards H_2_O_2_ to produce ROS. The subscript number on the noble metal (Au_6_NPs, Pd_6_NPs and Au_3_Pd_3_NPs) nanocatalysts refers to the mole ratio of the gold or palladium salt to 20 mg of CuONRs used. The preparation and characterization of CuONRs using chemical reduction method and deposition of nanocatalysts is to our knowledge reported here for the first time. The synthesized CuONORs and the adsorbed or deposited noble metal nanocatalysts were evaluated for their H_2_O_2_ catalytic reduction and production of reactive oxygen radical species (ROS). Owing to the superior catalytic activity of small size Au_6_NPs, Pd_6_NPs and the bimetallic Au_3_Pd_3_NPs and the catalytic activity of CuONRs, the CuONRs@Au_6_NPs, CuONRs@Pd_6_NPs, and CuONRs@Au_3_Pd_3_NPs could possess synergistic catalytic properties. The facile synthesis and low cost of CuONRs make them suitable catalyst support. Also, the novelty of this work is not limited to the synthesis of the nanoparticles alone. The evaluation of their peroxidase mimetic activity and application in glucose detection is also reported.

## Experimental

### Reagents and apparatus

Copper acetate Cu(Ac)_2_, hydrogen tetrachloroaurate trihydrate (HAuCl_4_·3H_2_O, 99%), palladium chloride (H_2_PdCl_4_), trisodium citrate dihydrate (Na_3_cit·2H_2_O), sodium borohydride (NaBH_4_), 3,3′,5,5′-tetramethylbenzidine (TMB), glacial acetic acid (CH_3_COOH), 1,3-diphenylisobenzofuran (DPBF), d-glucose powder, glucose oxidase (GOx, EC 1.1.3.4. from Aspergillus niger, Type VII) were purchased from Sigma-Aldrich. Potassium hydroxide pellets (KOH), sodium acetate anhydrous (NaAc), and potassium dihydrogen orthophosphate (KH_2_PO_4_) were purchased from Merck. Sodium hydroxide pellets (NaOH), absolute ethanol (EtOH), methanol (MeOH), 50% hydrogen peroxide (H_2_O_2_), 32% hydrochloric acid (HCl) and dimethyl sulfoxide (DMSO) were purchased from B&M Scientific. All the reagents were of analytical grade and were used as received from the suppliers. Ultrapure water with the resistivity of 18 MΩ cm obtained from a Milli-Q water system (Millipore Corp, Bedford, MA, USA) was used to prepare all aqueous solutions.

UV-visible measurements were conducted on a Thermo-Scientific, Multiskan Sky w Cuvette & Touch Screen, 100–240 V, Belgium. Zeta potential measurements were carried out on a Malvern Zetasizer Nano-ZS90 series equipped with a 633 nm He/Ne laser. A disposable folded capillary cell (DTS 1060) was used for data collection. TEM images were taken from a Zeiss Libra 120 TEM operating at 80 kV. The nanoparticles were dissolved in water and dropped onto a carbon-coated copper grid, and allowed to dry at room temperature overnight before data collection. The EDS spectra were obtained from INCA PENTA FET coupled with VGA TASCAM at 20 kV acceleration voltage. X-ray powder diffraction (XRD) patterns were recorded on a Bruker D8 Discover equipped with a Lynx Eye detector, using Cu-Kα radiation (1.5405 Å, nickel filter). The samples were placed on a silicon wafer slide. The data was collected within the 2*θ* range of 10° to 100°.

### Preparation of mono and bimetallic nanoparticles supported on CuONRs

#### Preparation of copper oxide nanorods (CuONRs)

The copper oxide nanorods were prepared following a reported sol–gel method^24^ with slight modifications. Briefly, 75 ml of 80 mM (6.0 mmol) copper acetate solution was blended with 1 ml glacial acetic acid in a round-bottom flask. The reaction was heated to reflux with continuous stirring. A 10 ml solution of sodium hydroxide (6 M, 60 mmol) was quickly injected into the solution. A black precipitate formed immediately. The reaction was allowed to stir for 30 minutes. The precipitate was centrifuged, washed three times with ethanol and air-dried at room temperature to yield copper oxide nanorods, CuONRs.

#### Preparation of AuNPs supported on CuONRs, CuONRs@Au_6_NPs

The preparation of CuONRs@Au_6_NPs was achieved using a method that has been previously reported.^20^ Briefly, 20 mg of copper oxide nanorods (CuONRs) was dispersed in 60 ml of Millipore water and sonicated for 30 minutes. Then 10 ml of HAuCl_4_·3H_2_O (0.60 mM, 6.0 mmol) and 1.5 ml of trisodium citrate dihydrate (3.0 mg, 0.010 mmol) were added to the CuONRs. After 15 minutes, 1.5 ml of sodium borohydride (3.0 mg, 0.080 mmol) was added to the solution. The colour changed immediately from dark brown to black. The reaction was stirred vigorously for another 30 minutes at room temperature. The black precipitate was centrifuged, washed three times with ethanol and air-dried at room temperature to yield gold nanoparticles-supported copper oxide nanorods, CuONRs@Au_6_NPs. The subscript number 6 refers to 6.0 mmol of HAuCl_4_·3H_2_O used.

#### Preparation of PdNPs supported on CuONRs, CuONRs@Pd_6_NPs

A similar procedure for CuONRs@Au_6_NPs was used to prepare CuONRs@Pd_6_NPs. Instead of HAuCl_4_·3H_2_O, H_2_PdCl_4_ salt was used. Briefly, 20 mg of copper oxide nanorods (CuONRs) was dispersed in 60 ml of Millipore water and sonicated for 30 minutes, and into this solution, 10 ml of H_2_PdCl_4_ (0.60 mM, 6.0 mmol) was added. About 1.5 ml of trisodium citrate dihydrate (3.0 mg, 0.010 mmol) and after 15 minutes, 1.5 ml of sodium borohydride (3.0 mg, 0.080 mmol) was added all at once. The colour changed immediately from dark brown to black. The reaction was stirred vigorously for another 30 minutes at room temperature. The black precipitate was centrifuged, washed three times with ethanol and air-dried at room temperature to yield palladium nanoparticles-supported CuONRs, CuONRs@Pd_6_NPs. The subscript number 6 refers to 6.0 mmol of H_2_PdCl_4_ used.

#### Preparation of AuPdNPs supported on CuONRS, CuONRs@Au_3_Pd_3_ NPs

The preparation of CuONRs@Au_3_Pd_3_NPs was accomplished by adding 20 mg of copper oxide nanorods (CuONRs) in 60 ml of water and sonicated for 30 minutes. Into this solution, HAuCl_4_·3H_2_O (5 ml, 3.0 mmol) and H_2_PdCl_4_ (5 ml, 3.0 mmol) aqueous solutions were added simultaneously at room temperature. Trisodium citrate dihydrate (3.0 mg, 0.010 mmol) was added to the mixture. After 15 minutes, sodium borohydride (3.0 mg, 0.080 mmol) was added rapidly into the solution. The colour changed immediately from dark brown to black. The reaction was stirred vigorously for 30 minutes at room temperature. The precipitate was centrifuged, washed three times with ethanol and air-dried at room temperature to yield bimetallic gold–palladium-supported copper oxide nanorods, CuONRs@Au_3_Pd_3_NPs.

### Monitoring catalytic reduction of H_2_O_2_

The catalytic reduction of H_2_O_2_ by gold and palladium mono- and bimetallic nanoparticles supported on CuONRs was conducted at room temperature using 3,3′,5,5′-tetramethyl benzidine (TMB) using UV-vis spectrophotometer. H_2_O_2_ (50 μL, 0.10 mol L^−1^), TMB (50 μL, 25 mmol L^−1^), and nanoparticle solution (50 μL of 2.0 mg mL^−1^) were added to 0.20 mol L^−1^ acetate buffer solution at the pH where IEP of the nanoparticles was zero. The blue colour developed with time, and the UV-vis spectra were measured.

In addition to the TMB, ROS generation was further monitored using 1,3-diphenyliso-benzofuran (DPBF). H_2_O_2_ (75 μL, 0.10 mmol L^−1^) was mixed with nanoparticles (50 μL, 2.0 mg mL^−1^). DPBF (50 μL, 0.25 mmol L^−1^) was added, and immediately the UV-vis spectra were measured. The absorption band at 430 nm was monitored. DPBF reacts with the produced ROS resulting in the oxidation of DPBF and a decrease in its signal intensity at 430 nm. The absorption spectra at 430 nm were obtained at a predetermined time interval.

### Detection of glucose at gold and palladium nanocatalysts supported on CuONRs

The ROS generation and optimal properties were evaluated towards the detection of glucose. The enzymatic reaction between glucose and glucose oxidase (GOx) to yield gluconolactone and H_2_O_2_ (by-product) was used. GOx (50 μL, 1.0 mg mL^−1^) was added into a 75 μL phosphate buffer saline (pH 7.4) solution of varied concentrations of glucose and allowed to react at 35 °C for 45 minutes. This results in the oxidation of glucose to produce gluconolactone and H_2_O_2_. After 45 minutes, 50 μL solution (2.0 mg mL^−1^) of gold and palladium-supported nanorods was added. This was followed by the addition of TMB (100 μL, 2.0 mmol L^−1^) in acetate buffer (1.0 ml, 0.20 mol L^−1^) at optimum pH. The temperature of the solution was kept at 35 °C. The colour development was monitored by the naked eye (blue colour development) and spectrophotometrically using UV-vis.

## Results and discussion

### Characterisation of gold- and palladium-supported CuONRs

CuONRs nanoparticles were prepared using the chemical reduction method.^20^ The conventional sol–gel method was optimised to yield CuO nanorods (CuONRs). The rod-like morphology of CuONRs provided a suitable support surface for anchoring gold and palladium nanoparticles. A seed-mediated method was used to deposit gold and palladium nanoparticles and played an essential role in the formation CuONRs@Au_6_NPs, CuONRs@Pd_6_NPs and CuONRs@Au_3_Pd_3_NPs. The preparation of gold and palladium nanoparticles-supported on CuONRs was conducted in three separate reaction vessels.

Into each reaction vessel, CuONRs was dispersed in solution, and noble metal salt HAuCl_4_·3H_2_O or H_2_PdCl_4_ or a mixture of both was added. After 15 minutes of homogeneously mixing the reaction, a strong reducing agent NaBH_4_) was added. Trisodium citrate was added as the stabilising agent. The concentrations of metal salts and NaBH_4_ were such that small noble metal nanoparticles were deposited onto CuONRs. The reaction was carried out in an ice-water bath. The low temperatures were used to facilitate the formation of smaller size gold and palladium nanoparticles onto the CuONRs surface. For metal salts, 0.60 mmol L^−1^ concentration was used, and for mixed metal salts, a concentration of 0.30 mmol L^−1^ for each metal salt was used. The formation of CuONRs@Au_6_NPs and CuONRs@Pd_6_NPs occurred and represented monometallic supported nanoparticles. Bimetallic gold–palladium nanoparticles involved simultaneous co-reduction of HAuCl_4_·3H_2_O (0.30 mM) and H_2_PdCl_4_ (0.30 mM) to form CuONRs@Au_3_Pd_3_NPs. The morphology and structural properties were studied to understand the synergistic effect between metallic systems.^25^

**Fig. 1:**
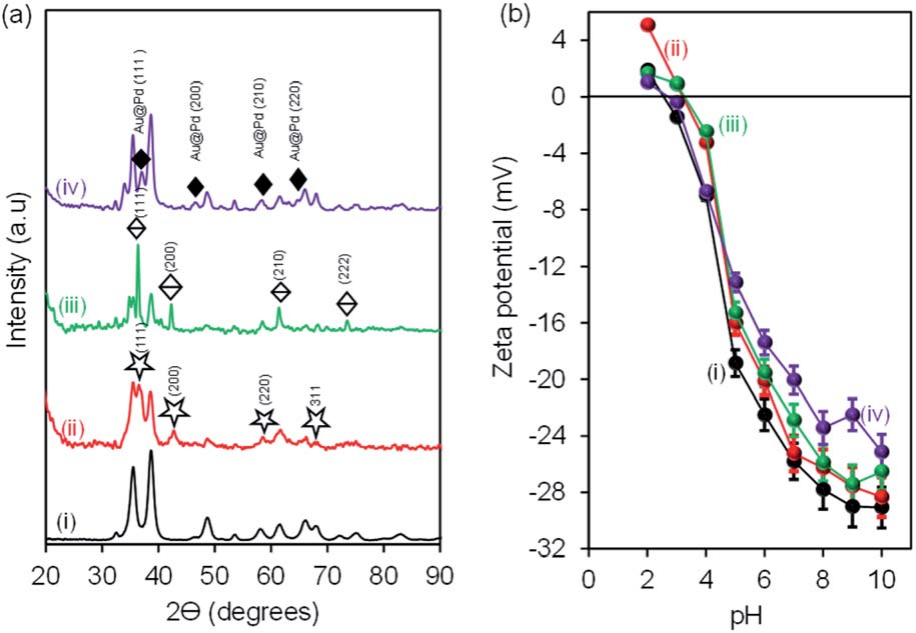
(a) X-ray diffraction (XRD) patterns and (b) zeta potential *vs.* pH of (i) CuONRs, (ii) CuONRs@Au_6_NPs, (iii) CuONRs@Pd_6_NPs, and (iv) CuONRs@Au_3_Pd_3_NPs.

X-ray diffraction (XRD) was used to analyse the successful formation of CuONRs and mono and bimetallic gold and palladium nanoparticles supported on CuONRs. Fig. 1 shows the (a) X-ray diffraction patterns and (b) zeta-potentials of (i) CuONRs, (ii) CuONRs@Au_6_NPs, (iii) CuONRs@Pd_6_NPs, and (iv) CuONRs@Au_3_Pd_3_NPs. The X-ray diffractogram for CuONRs in Fig. 1(a)(i) exhibited peaks at 2*θ* = 32.6°, 35.6°, 38.8°, 48.0°, 53.3°, 57.9°, 61.5°, 65.9°, 67.5°, 71.9°, 74.5° corresponding to (110), (−111), (111), (−202), (020), (202), (−113), (022), (311), (004) Miller indices. The X-ray diffraction patterns showed single phase copper oxide nanoparticles with a monoclinic structure with lattice constants, *a* = 4.67970, *b* = 3.43140, *c* = 5.13620, *α* = 90.00, *β* = 99.26 and *γ* = 90.00. The peaks were broad in shape, confirming the formation of the nanoparticles. The CuONRs@Au_6_NPs in Fig. 1(a)(ii) showed the diffraction patterns due to CuONRs as observed above and additional peaks at 2*θ* = 37.4°, 44.4°, 64.5°, 77.7° corresponding to (111), (200), (220), (311) Miller indices of gold nanoparticles similar to a face-centred cubic crystal structure of gold (JCPDS: 65-8601). The CuONRs@Pd_1.0_NPs in Fig. 1(a)(iii) showed the diffraction patterns due to CuONRs and PdNPs at 2*θ* = 40.1°, 46.3°, 68.5°, 82.1°, 86.0° corresponding to (111), (200), (220), (311), (222) Miller indices of PdNPs similar to the structure of palladium (JCPDS: 05-0681). The CuONRs@Au_3_Pd_3_NPs in Fig. 1(a)(iv) showed the mixture of diffraction patterns of CuONRs, CuONRs@Au_6_NPs and CuONRs@Pd_6_NPs. There were also few additional peaks at 2*θ* = 34.1, 38.2, 63.8 and 82.7°, corresponding to (111), (200), (220), and (311) for the nanoalloy of Au@PdNPs. The distinct diffraction patterns emanating from CuONRs@Au_3_Pd_3_NPs confirmed co-reduction of HAuCl_4_·3H_2_O and H_2_PdCl_4_ salts occurred, and the formation of Au@PdNPs nanoalloy formed successful.^4^Fig. 1(b) shows the zeta potential (mV) plot of the synthesised nanomaterials at different pH conditions. The zeta potential graph showed similar trends with the zeta potential values positive at pH 2 for CuONRs and CuONRs@Au_6_NPs. For CuONRs@Pd_6_NPs and CuONRs@Au_3_Pd_3_NPs, the values were positive at pH 2 and pH 3. The zeta potential values were negative and continued to increase negatively as the pH increased. This is due to hydroxide (OH^−^) from the sodium hydroxide salt used to prepare CuONRs. In addition to the OH^−^, also carboxylic acid and hydride surface functional groups due to adsorbed species which act as stabilisers of gold and palladium nanoparticles. The negative increase is due to the deprotonation of the surface carboxyl groups as the pH increases. The zeta potential values increased to −29.1 mV for CuONRs, −28.3 mV for CuONRs@Au_6_NPs, −27.4 mV for CuONRs@Pd_6_NPs and −25.1 mV for CuONRs@Au_3_Pd_3_NPs at pH 10. The negative zeta potentials for CuONRs@Au_6_NPs, CuONRs@Pd_6_NPs and CuONRs@Au_3_Pd_3_ NPs were ascribed to the citrate stabiliser used to minimise aggregation of Au and Pd nanoparticles on the surface of CuONRs. The negative zeta potential was also due to the exposed CuONRs surface not covered by Au and Pd nanoparticles. The CuONRs exhibited the isoelectric point (IEP) or point-of-zero charge (PZC) at pH 2.5. The IEP was slightly shifted for CuONRs@Au_6_NPs to pH 3.2, CuONRs@Pd_6_NPs to pH 3.3 and CuONRs@Au_3_Pd_3_NPs to pH 2.7. High positive (>+20 mV) or negative (>−20 mV) zeta potential values are related to the stability of the nanoparticles at that pH as these induce particle–particle separation due to charge repulsion.^26^ The zeta potential values more than −20 mV for stable nanoparticles were at pH ≥ 5 for CuONRs, pH ≥ 6 for CuONRs@Au_6_NPs, pH ≥ 6 for CuONRs@Pd_6_NPs and pH ≥ 7 for CuONRs@Au_3_Pd_3_NPs. The negative zeta potential shows that the mono and bimetallic nanoparticles supported on CuONRs are negatively charged and stable at physiological pH.

**Fig. 2:**
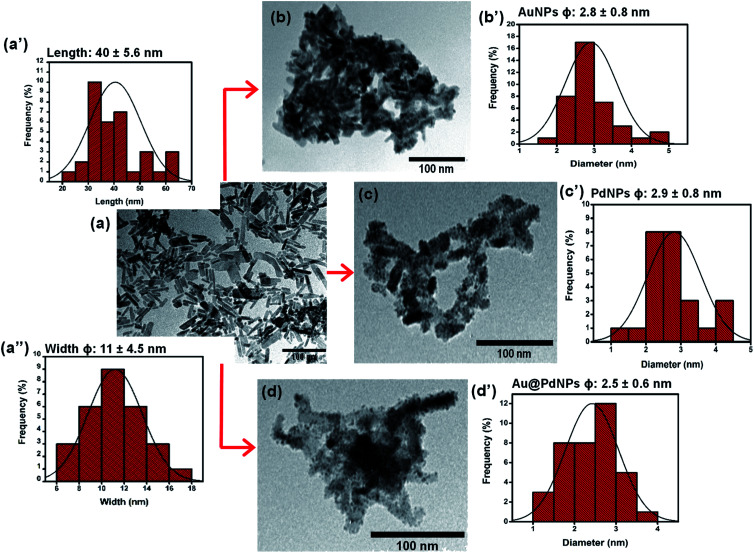
TEM micrographs with their corresponding size distribution histograms and their corresponding for (a) CuONRs, (b) CuONRs@Au_6_NPs, (c) CuONRs@Pd_6_NPs, and (d) CuONRs@Au_3_Pd_3_NPs.

Fig. 2 shows TEM micrographs with their corresponding size distribution histograms for (a) CuONRs, (b) CuONRs@Au_6_NPs, (c) CuONRs@Pd_6_NPs, and (d) CuONRs@Au_3_Pd_3_NPs. The TEM image of CuONRs in Fig. 2(a) exhibited rod-like nanostructures with the length of 40 ± 5.6 nm and the width (*ϕ*) of 11 ± 4.5 nm. The CuONRs TEM image also showed some aggregation, which is typical of copper oxide nanoparticles.^24^ The bigger-sized nanoparticles were due to the overlapping CuONRs, and the longer CuONRs were head-to-tail or head-to-head or tail-to-tail connected. The CuONRs that overlapped or head-to-tail or head-to-head or tail-to-tail connected were not included in measuring the length (40 ± 5.6 nm) and width (11 ± 4.5 nm). The TEM image of CuONRs@Au_6_NPs in Fig. 2(b) showed that the rod-like morphology of CuONRs was retained, but these were clustered. Spherical gold nanoparticles were observed as dark spots deposited onto CuONRs. The deposited AuNPs onto CuONRs resulted in the rough surfaces of CuONRs. The average particle size of AuNPs on the surface of CuONRs was 2.8 ± 0.8 nm. The TEM image of CuONRs@Pd_6_NPs exhibited spherical palladium nanoparticles as deposits on the surface of CuONRs. The average size of palladium nanoparticles was 2.9 ± 0.8 nm. The gold and palladium nanoparticles gave similar particle sizes, and this was due to similar concentrations (0.60 mmol L^−1^) of HAuCl_4_·3H_2_O and H_2_PdCl_4_ used and similar reaction conditions. The average size of AuPd bimetallic nanoparticles on the surface of CuONRs was 2.5 ± 0.6 nm. The relatively smaller size nanoparticles of AuPdNPs was due to the concentration (0.30 mmol L^−1^) used for each metal salt. The total metal salt content was 0.60 mmol L^−1^ and equivalent to the CuONRs@Au_6_NPs and CuONRs@Pd_6_NPs. The SEM-EDS was used to characterise the nanoparticles, and the results are shown in Fig. S2 to S4 of the (ESI†). The SEM-EDS elemental mapping showed the homogeneous and uniform distribution of copper, oxygen, gold and palladium in the prepared nanoparticles.

### Catalytic reduction of H_2_O_2_ at Au_6_NPs-, Pd_6_NPs- and Au_3_Pd_3_NPs-supported on CuONRs

**Fig. 3:**
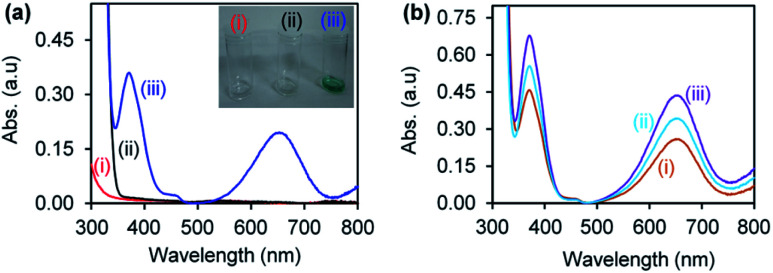
UV-vis absorption spectra of (a)(i) NRs + H_2_O_2_, (ii) NRs + TMB, (iii) CuONRs + H_2_O_2_ + TMB and (b)(i) CuONRs@Au_6_NPs + H_2_O_2_ + TMB, (v) CuONRs@Pd_6_NPs + H_2_O_2_ + TMB, (vi) CuONRs@Au_3_Pd_3_NPs + H_2_O_2_ + TMB in 0.20 M acetate buffer solution.

Gold and palladium nanoparticles supported on CuONRs were investigated for their catalytic reduction of H_2_O_2_ to form ROS (HO˙^−^, HO_2_˙^−^ and O_2_˙^−^). The reaction was monitored following the oxidation of a chromogenic substrate, 3,3′,5,5′-tetramethyl-benzidine (TMB). TMB is a colourless chromogenic substrate. Upon oxidation by ROS, it changes colour to blue, resulting in a 3,3′,5,5′-tetramethylbenzidinediimine (TMBDI) charge transfer complex with a maximum absorption bands at 652 nm and 370 nm. Fig. 3 shows the UV-vis absorption spectra of (a)(i) nanomaterials (Au_6_NPs-, Pd_6_NPs- and Au_3_Pd_3_NPs-supported on CuONRs) + H_2_O_2_, (ii) nanomaterials + TMB, and (iii) CuONRs + H_2_O_2_ + TMB (inset: images of corresponding solutions), and (b)(i) CuONRs@Au_6_NPs + H_2_O_2_ + TMB, (ii) CuONRs@Pd_6_NPs + H_2_O_2_ + TMB, (iii) CuONRs@Au_3_ Pd_3_NPs + H_2_O_2_ + TMB in 0.20 M acetate buffer solution (at the pH where IEP is zero for different nanoparticles). No colour development and absorption in the UV-vis spectra were observed in Fig. 3(a)(i) and (ii). Fig. 3(a)(iii) shows the UV-vis spectrum with absorption maxima at 370 nm and 652 nm due to the presence of H_2_O_2_ + TMB + CuONRs. The images of the different solutions in the inset of Fig. 3(a)(iii) showed that blue colour developed. The UV-vis spectra in Fig. 3(b)(i) CuONRs@Au_6_-NPs, (ii) CuONRs@Pd_6_NPs and (iii) CuONRs@Au_3_Pd_3_NPs showed absorption with maxima at 370 nm and 652 nm. The observed absorption bands at 370 nm and 652 nm confirmed ROS generation from the reduction of H_2_O_2_ and oxidation of TMB. The oxidation of TMB only occurs when the nanoparticles are in the presence of H_2_O_2_.

### Optimum conditions for H_2_O_2_ reduction and ROS generation

**Fig. 4:**
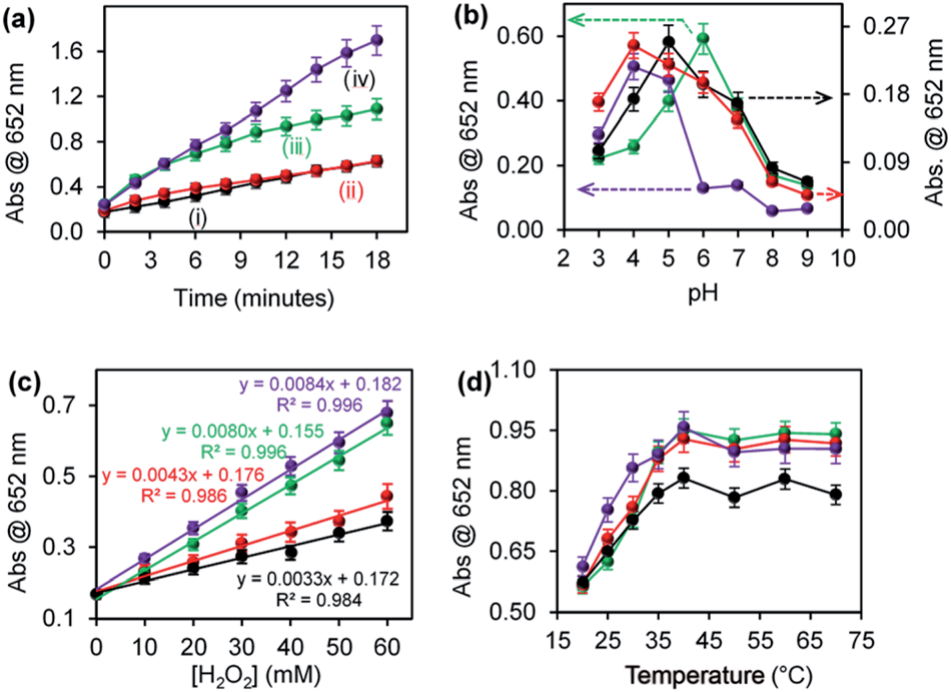
Effect of (a) reaction time, (b) pH, (c) changing H_2_O_2_ concentration, and (d) temperature on peroxidase-like activity of (i) CuONRs (black), (ii) CuONRs@Au_6_NPs (red), (iii) CuONRs@Pd_6_NPs (green), and (iv) CuONRs@Au_3_Pd_3_NPs (purple).

The effect of environmental conditions such as pH, H_2_O_2_ concentration and temperature on the ROS generation efficiency were investigated. Fig. 4 shows the effect of (a) reaction time, (b) pH, (c) varying H_2_O_2_ concentration, and (d) temperature on ROS generation for (i) CuONRs, (ii) CuONRs@ Au_6_NPs (red), (iii) CuONRs@Pd_6_NPs (green), and (iv) CuONRs@Au_3_Pd_3_NPs (purple). In Fig. 4(a), the effect of reaction time was investigated by increasing the reaction time from 0–18 minutes. The CuONRs@Au_3_Pd_3_NPs in Fig. 4(a)(iv) showed the highest rate of ROS generation compared to other nanoparticles. CuONRs and CuONRs@Au_6_NPs showed the lowest ROS generation in Fig. 4(a)(i) and (ii), respectively. CuONRs@Pd_6_NPs in Fig. 4(a)(iii) showed higher ROS generation when compared with CuONRs and CuONRs@Au_6_NPs. In Fig. 4(b), the effect of pH was investigated by changing the pH from pH 3–9 of the substrate solution. ROS generation was highest at pH 4.0 for CuONRs@Au_6_NPs and CuONRs@Au_3_Pd_3_NPs, pH 5.0 for CuONRs, and pH 6.0 for CuONRs@Pd_6_NPs. The differences in optimum pH conditions were attribute to variation in the composition of the prepared nanomaterials. It could be inferred that CuONRs@Au_6_NPs and CuONRs@Au_3_Pd_3_NPs favoured ROS generation in pH 4.0, whilst CuONRs favoured ROS generation at pH 5.0 with CuONRs@Pd_6_NPs favouring ROS generation at pH 6.0. As the pH increased, a decrease in ROS generation was observed due to the instability and decomposition of H_2_O_2_ in alkaline conditions. In Fig. 4(c), the effect of varying H_2_O_2_ concentration from 0–60 mM was investigated. The concentration of TMB was kept constant (25 mM). When the H_2_O_2_ concentration increased from 0–60 mM, ROS generation increased for CuONRs, CuONRs@Au_6_NPs, CuONRs@Pd_6_NPs and CuONRs@Au_3_Pd_3_NPs. A linear relationship between the absorbance at 652 nm and TMB or H_2_O_2_ concentration was established. CuONRs@Au_3_Pd_3_NPs had the highest slope of 0.0084 a.u. mM^−1^ followed by CuONRs@Pd_6_NPs (0.0080 a.u. mM^−1^) and CuONRs @Au_6_NPs (0.0043 a.u. mM^−1^) and CuONRs (0.0033 a.u. mM^−1^). The high slope for CuONRs@Au_3_Pd_3_NPs was due to the excellent catalysis of the bimetallic system. In Fig. 4(d), the effect of increasing the temperature from 20–70 °C was investigated. The studied nanomaterials showed an increase in ROS generation as the temperature increased up to 40 °C and remained stable afterwards. It was interesting to see that ROS generation for the nanomaterials was not affected by the temperature up to 70 °C. The stability of the ROS generation even at increased temperatures demonstrates the applicability of the nanomaterials in temperature conditions unfavourable to natural enzymes. In terms of the nanomaterials used, CuONRs@Au_3_Pd_3_NPs showed higher absorption intensity as the reaction time increased, and this was followed by the CuONRs@Pd_6_NPs. This could be attributed to the excellent catalytic properties of PdNPs in CuONRs@Pd_6_NPs. The bimetallic nanoparticle system, CuONRs@Au_3_Pd_3_NPs, containing AuNPs and PdNPs as nanocatalysts resulted in the synergistic effect and higher rate of ROS generation as confirmed by an enhanced increase in absorption at 652 nm.

### Steady-state kinetics of Au_6_NPs-, Pd_6_NPs- and Au_3_Pd_3_NPs-supported on CuONRs

**Fig. 5:**
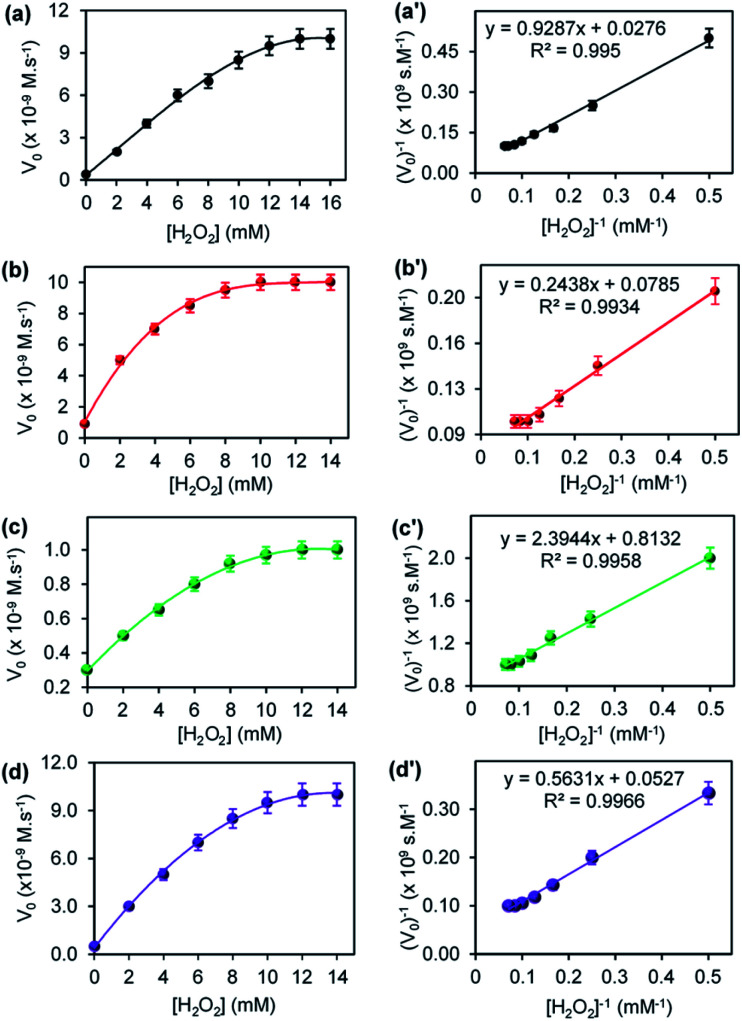
The steady-state kinetic plots of varying [H_2_O_2_] at a fixed 25.0 mM TMB concentration and their corresponding double reciprocal plots for (a) CuONRs, (b) CuONRs@Au_6_NPs, (c) CuONRs@Pd_6_NPs and (d) CuONRs@Au_3_Pd_3_NPs.

The steady-state kinetic parameters were evaluated for CuONRs and Au_6_NPs-, Pd_6_NPs-, and Au_3_Pd_3_NPs supported on CuONRs. The Michaelis–Menten and Lineweaver–Burk (double reciprocal) models were used to obtain the kinetic parameters derived from varying one substrate concentration and keeping the other substrate concentration constant. H_2_O_2_ and TMB were used as substrates. The Michaelis–Menten parameters such as Michaelis–Menten constant (*K*_m_) and maximum velocity (*V*_max_) were obtained by fitting the data obtained from the plot of initial velocity (*V*_o_) *versus* substrate concentration [*S*] in the Lineweaver–Burk double reciprocal plot. Fig. 5 shows a plot of initial velocity (*V*_o_) *versus* varied [H_2_O_2_] at a fixed 25.0 mM of [TMB], and the corresponding double reciprocal plots for (a) CuONRs, (b) CuONRs@Au_6_NPs, (c) CuONRs@Pd_6_NPs and (d) CuONRs@Au_3_Pd_3_NPs. The linear relationship between initial velocity (*V*_o_) *versus* [H_2_O_2_] was observed at low [H_2_O_2_] concentrations and a plateau at higher [H_2_O_2_] concentrations confirming Michaelis–Menten kinetics. The Lineweaver–Burk (double reciprocal plots) were used to calculate *V*_max_ values from the intercepts and the slopes of the graph for calculating *K*_m_ values. The *K*_m_ and *V*_max_ values obtained are summarised in Table 1 for H_2_O_2_ and TMB as substrates. The *K*_m_ value indicates the binding affinity of the nanoparticles towards the substrate.^27,28^ Low *K*_m_ values confirm a stronger binding affinity, while higher *K*_m_ values signify a weak binding affinity towards a particular substrate. The CuONRs@Pd_6_NPs showed a lowest *K*_m_ value of 2.94 mM, whilst CuONRs@Au_6_NPs showed the highest *K*_m_ value of 3.11 mM when H_2_O_2_ was used as a substrate. The results indicate that the CuONRs@Pd_6_NPs had a stronger binding affinity for H_2_O_2_. CuONRs@Au_3_Pd_3_NPs exhibited a high *K*_m_ value of 10.68 mM compared to CuONRs@Au_6_NPs (3.11 mM) and CuONRs@Pd_6_NPs (2.94 mM). However, the *K*_m_ value for CuONRs@Au_3_Pd_3_NPs of 10.68 mM was lower than 39.97 mM of CuONRs, confirming that Au_3_Pd_3_NPs had an enhancing effect. The *K*_m_ values for CuONRs@Au_6_NPs and CuONRs@Pd_6_NPs were within the *K*_m_ values for the HRP enzyme, ranging between 0.214–3.72 mM. The following trend of increasing *K*_m_ values was observed; CuONRs@Pd_6_NPs (2.94 mM) < CuONRs@ Au_6_NPs (3.11 mM) < CuONRs@Au_3_Pd_3_NPs (10.68 mM) < CuONRs (39.97 mM). The excellent *K*_m_ values for CuONRs@Pd_6_NPs confirms the observed trend on the enhancement of ROS generation and due to electron transfer from gold to palladium as observed above.

**Table 1:** Steady-state kinetic parameters, *K*_m_ and *V*_max_ for the AuNPs, PdNPs and AuPdNPs-supported CuONRs

Material	Substrate	*K*_m_ (mM)	*V*_max_ (M s^−1^)
CuONRs^TW^	[H_2_O_2_]	39.97	4.24 × 10^−8^
	[TMB]	2.79	1.55 × 10^−8^
CuONRs@Au_6_NPs^TW^	[H_2_O_2_]	3.11	1.27 × 10^−8^
	[TMB]	6.34	2.44 × 10^−8^
CuONRs@Pd_6_NPs^TW^	[H_2_O_2_]	2.94	1.23 × 10^−9^
	[TMB]	3.74	1.65 × 10^−8^
CuONRs@Au_3_Pd_3_NPs^TW^	[H_2_O_2_]	10.68	1.90 × 10^−8^
	[TMB]	4.55	7.25 × 10^−9^
HRP^21,26^	[H_2_O_2_]	0.214–3.72	1.24 × 10^−8^ to 8.71 × 10^−8^
	[TMB]	0.275–0.434	2.46 × 10^−8^ to 10.0 × 10^−8^
CuO–Au nanoalloy^30^	[H_2_O_2_]	4.08	1.11 × 10^−10^
	[TMB]	3.54	1.05 × 10^−10^
Fe_3_O_4_@SiO_2_–NH_2_–Au@Pd_0.30_NPs^31^	[H_2_O_2_]	0.35	6.78 × 10^−8^
	[TMB]	0.09	8.65 × 10^−8^

**Fig. 6:**
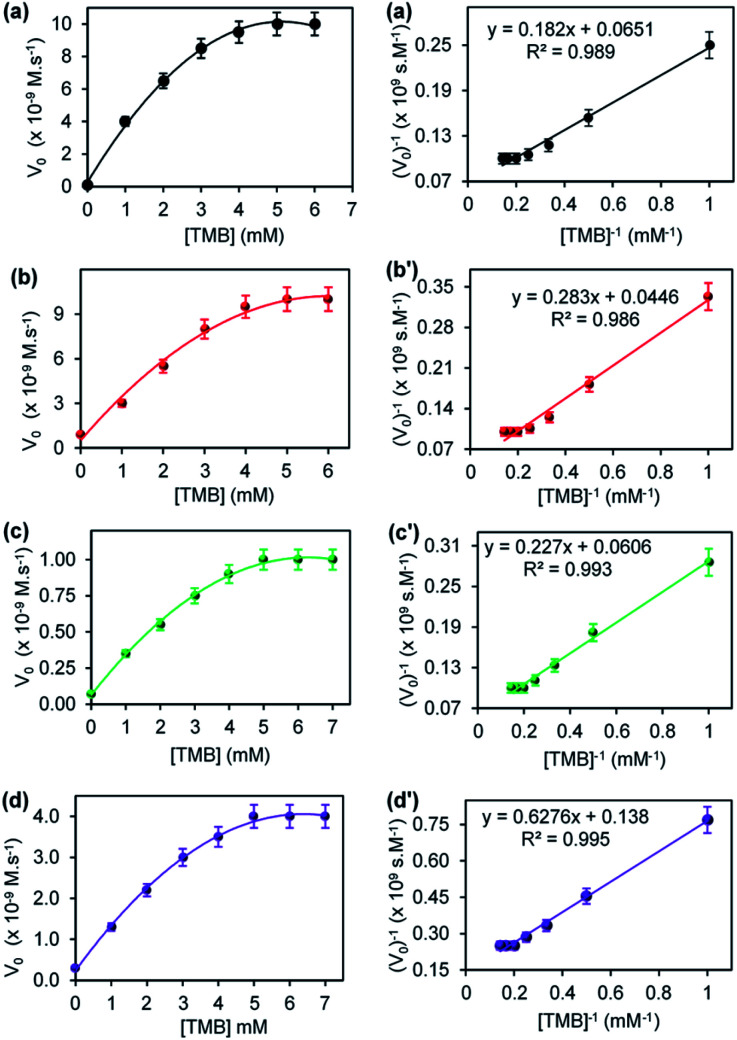
The steady-state kinetic plots of varying TMB concentrations at a fixed 0.10 M H_2_O_2_ concentration and the corresponding double reciprocal plots for (a) CuONRs, (b) CuONRs@Au_6_NPs, (c) CuONRs@Pd_6_NPs, and (d) CuONRs@Au_3_Pd_3_NPs.

Fig. 6 shows the steady-state kinetic parameters, plot of initial velocity (*V*_o_) *versus* [TMB], and their corresponding double reciprocal plots for (a) CuONRs, (b) CuONRs@Au_6_NPs, (c) CuONRs@Pd_6_NPs and (d) CuONRs@Au_3_Pd_3_NPs. The TMB concentration was varied from 0–7.0 mM whilst keeping the concentration of H_2_O_2_ (0.10 M) constant for the different nanomaterials. At lower TMB concentrations (<5.0 mM), the initial velocity (*V*_o_) increased linearly with increasing concentration of TMB for CuONRs in Fig. 6(a), CuONRs@Au_6_NPs Fig. 6(b), CuONRs@Pd_6_NPs in Fig. 6(c) and CuONRs@Au_3_Pd_3_NPs in Fig. 6(d) and reaching the plateau afterwards. The obtained results are representative of the Michaelis–Menten mechanism. The *K*_m_ and *V*_max_ values were calculated and are summarised in Table 1 for both H_2_O_2_ and TMB as substrates. The CuONRs showed a strong binding affinity towards TMB with *K*_m_ equals to 2.79 mM followed by CuONRs@Pd_6_NPs with a *K*_m_ value of 3.74 mM. The *K*_m_ values for CuONRs@Au_6_NPs and CuONRs@Au_3_Pd_3_ NPs were 4.55 mM and 6.34 mM, respectively. The trend of increasing *K*_m_ values was CuONRs (2.79 mM) < CuONRs@Pd_6_NPs (3.74 mM) < CuONRs@Au_3_Pd_3_NPs (4.55 mM) < CuONRs@Pd_6_NPs (6.34 mM). The maximum velocity (*V*_max_) for TMB was 7.25 × 10^−9^ M s^−1^ for CuONRs@Au_3_Pd_3_NPs, whilst that of other materials was 10^−8^ M s^−1^. The *K*_m_ values of TMB for the prepared nanomaterials were higher than for HRP enzyme towards TMB with *K*_m_ values 0.275–0.434 mM (ref. 21 and 29) and for Au@Pd_0.30_NPs supported on magnetic–silica nanoparticles.^30^ The *K*_m_ values were comparable to the reported CuO–Au nanoalloys recently reported.^31^ CuONRs@Pd_6_NPs exhibited better kinetic properties, especially towards H_2_O_2_, which is the substrate that interacts with the nanomaterial and generates ROS that oxidises the TMB substrate.

### Confirmation of ROS generation using DPBF

**Fig. 7:**
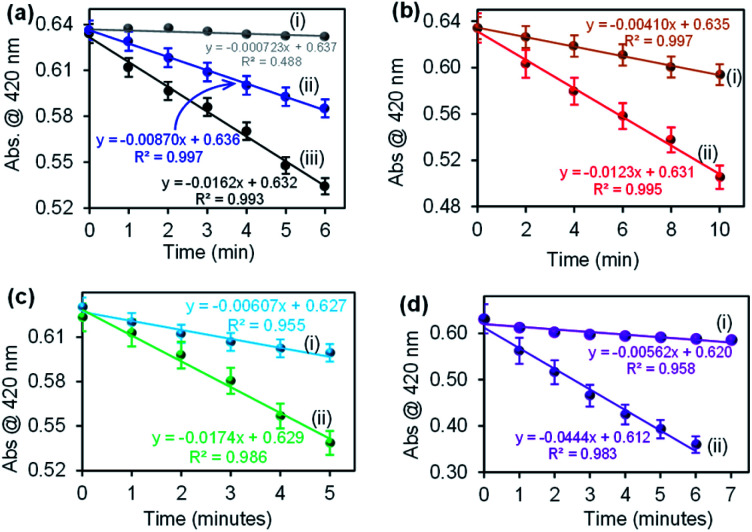
The rate of oxidation plots for (a)(i) DPBF + H_2_O_2_, (ii) DPBF + CuONRs and (iii) DPBF + H_2_O_2_ + CuONRs, (b) CuONRs@Au_6_NPs with (i) DPBF alone and (ii) DPBF + H_2_O_2_, (c) CuONRs@Pd_6_NPs (i) DPBF alone and (ii) DPBF + H_2_O_2_, and (d) CuONRs@Au_3_Pd_3_NPs with (i) DPBF alone and (ii) DPBF + H_2_O_2_. DPBF and (ii) DPBF + H_2_O_2_.

The catalysis of CuONRs, CuONRs@Au_6_NPs, CuONRs@Pd_6_NPs and CuONRs@Au_3_Pd_3_NPs was proposed to be *via* the reduction of H_2_O_2_ to produce ROS (OH˙^−^, HO_2_˙^−^ and O_2_˙^−^). The generation of ROS was evaluated using 1,3-diphenylisobenzofuran (DPBF) as a radical scavenger. DPBF is a fluorescent dye that reacts in a specific manner with singlet oxygen (^1^O_2_), leading to its oxidation, monitored using UV-vis spectroscopy.^32^ In this work, DPBF was used to monitor the production of ROS and its oxidation. The Au_6_NPs-, Pd_6_NPs- and Au_3_Pd_3_NPs supported on CuONRs produced radicals (HO˙^−^, HO_2_˙^−^, O_2_˙^−^) upon interacting with H_2_O_2_. Spectroscopically, the produced radicals were scavenged by DPBF, and a decrease in its absorption peak was observed. The rate of oxidation of DPBF was monitored at 1 or 2 minutes time intervals for the prepared nanoparticles. Fig. 7 shows the rate of oxidation plots for (a)(i) DPBF + H_2_O_2_, (ii) DPBF + CuONRs and (iii) DPBF + H_2_O_2_ + CuONRs, (b) CuONRs@Au_6_NPs (i) with DPBF alone and (ii) with DPBF + H_2_O_2_, (c) CuONRs@Pd_6_NPs with (i) DPBF alone and (ii) DPBF + H_2_O_2_ and (d) CuONRs@Au_3_Pd_3_NPs with (i) DPBF alone and (ii) DPBF + H_2_O_2_. There was a slight change in the absorption intensity for DPBF + H_2_O_2_, and the rate of oxidation of DPBF was 7.23 × 10^−4^ a.u. min^−1^. A slight decrease in DPBF absorption was observed in the presence of CuONRs, with the rate of oxidation increasing to 8.70 × 10^−3^ a.u. min^−1^. The rate of oxidation of DPBF was increased further when H_2_O_2_ and nanoparticles were all present in the solution. The rate of oxidation of DPBF was obtained as a slope of the linear plot of absorbance (a.u.) *vs.* time (min). The rates of oxidation for CuONRs, CuONRs@Au_6_NPs, CuONRs@Pd_6_NPs and CuONRs@Au_3_Pd_3_NPs are summarised in Table 2. The rate of oxidation of DPBF was faster in the presence of H_2_O_2_ and CuONRs@Au_3_Pd_3_NPs (4.44 × 10^−2^ a.u. min^−1^). The higher the rate of DPBF oxidation, the higher the concentration of ROS produced. The rate of oxidation of DPBF is equivalent to the rate of ROS produced and it was found to decrease following this trend: CuONRs@Au_3_Pd_3_NPs (4.44 × 10^−2^ a.u. min^−1^) > CuONRs@Pd_6_NPs (1.74 × 10^−2^ a.u. min^−1^) > CuONRs (1.62 × 10^−2^ a.u. min^−1^) > CuONRs@Au_6_NPs (1.23 × 10^−2^ a.u. min^−1^). The DPBF studies confirmed ROS production from the interaction of H_2_O_2_ with the nanomaterials and the highest ROS generation efficiency of CuONRs@Au_3_Pd_3_NPs. ROS generation from the H_2_O_2_ was an important first step towards the oxidation of TMB and not observed in the absence of H_2_O_2_. The mechanistic steps involved in H_2_O_2_ catalytic reduction are as follows: step 1 adsorption of H_2_O_2_*via* oxygen atoms onto the metal surfaces (Pd, Au and Cu) of the nanoparticle. Step 2 is the catalytic reduction which leads to bond breaking to produce ROS (OH˙^−^, HO_2_˙^−^ and O_2_˙^−^). Step 3, the generated ROS diffuses back into solution for further reaction. The noble metal nanoparticles (Au_6_NPs, Pd_6_NPs and Au_3_Pd_3_NPs) were adsorbed onto CuONRs *via* the lattice oxygen atoms. CuONRs@Pd_6_NPs and CuONRs@Au_3_Pd_3_NPs exhibited enhanced catalytic activity due to the presence of Pd nanoparticles.

**Table 2:** Comparison of the rate of oxidation of DPBF for the prepared Au_6_NPs, Pd_6_NPs and Au_3_Pd_3_NPs-supported on CuONRs

Nanomaterials	Rate of oxidation (min^−1^)		
	DPBF + H_2_O_2_ (×10^−4^)	DPBF (×10^−3^)	DPBF + H_2_O_2_ (×10^−2^)
CuONRs	7.23	8.70	1.62
CuONRs@Au_6_NPs		4.10	1.23
CuONRs@Pd_6_NPs		6.07	1.74
CuONRs@Au_3_Pd_3_NPs		5.62	4.44

### Glucose detection at Au_6_NPs-, Pd_6_NPs- and Au_3_Pd_3_NPs-supported on CuONRs

**Scheme 1:**
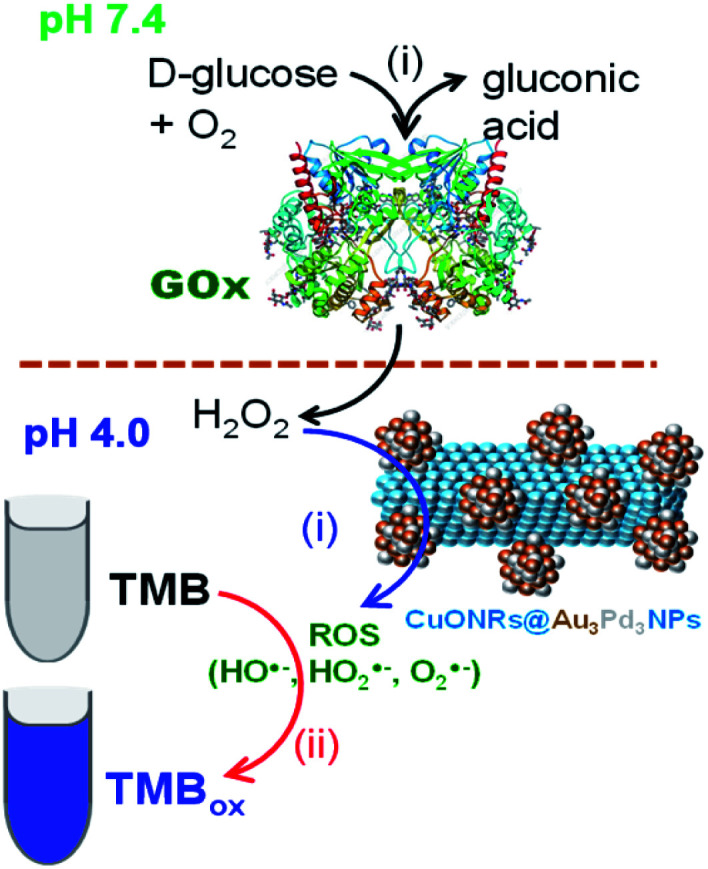
The mechanism of glucose detection using glucose oxidase (GOx) enzyme and CuONRs@Au_3_Pd_3_NPs as nanocatalyst for the catalytic reduction of H_2_O_2_ to produce ROS.

Glucose detection was investigated as the model analyte to demonstrate the applicability of the nanomaterials. Au_6_NPs, Pd_6_NPs and Au_3_Pd_3_NPs-supported on CuONRs were used in the colorimetric detection of glucose. H_2_O_2_ is the by-product of various oxidase enzymes with FADH_2_ enzyme co-factor such as glucose oxidase (GOx), alcohol oxidase (AOx), uric oxidase (UOx), and cholesterol oxidase (ChOx).^33^ GOx is used in the fabrication of biosensors for the detection of glucose. GOx enzymes as biocatalysts offer excellent selectivity and sensitivity towards the β-d-glucose substrates. After the production, H_2_O_2_ is catalytically oxidised at the nanoparticle surface and generate ROS, as confirmed above. In the presence of TMB as a chromogenic substrate, the produced ROS oxidises TMB to form blue coloured products (TMBDI) characterised by the absorption band at 652 nm in the UV-vis spectrum. The mechanism of this biocatalysis is shown in Scheme 1.

**Fig. 8:**
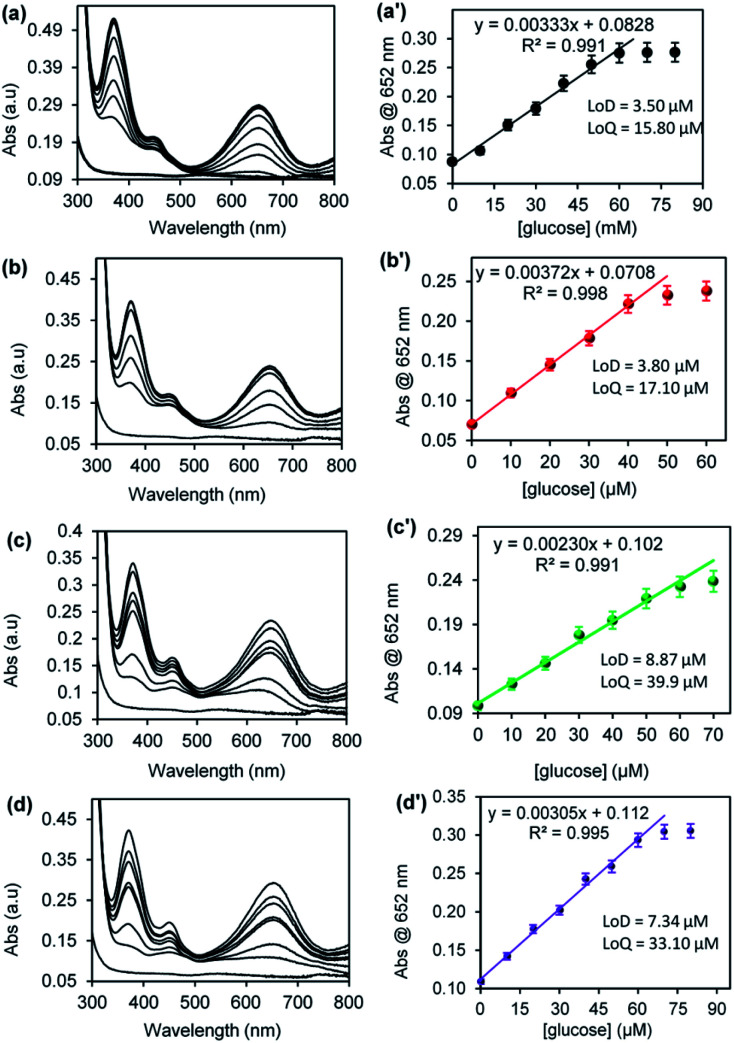
UV-vis spectra and the corresponding plots of absorbance @ 652 nm *versus* [glucose] concentration for (a) CuNRs, (b) CuONRs@Au_6_NPs, (c) CuONRs@Pd_6_NPs and (d) CuONRs@Au_3_Pd_3_NPs.

Fig. 8 shows the UV-vis spectra and the corresponding plots of the absorbance at 652 nm *versus* concentration of glucose for (a) CuONRs, (b) CuONRs@Au_6_NPs, (c) CuONRs@Pd_6_NPs, and (d) CuONRs@Au_3_Pd_3_NPs. The concentration of glucose was varied from 0–60 μM, whilst the concentration of GOx, nanomaterials, TMB and reaction time were kept constant. The UV-vis absorption at 652 nm increased linearly as the glucose concentration increases up to 40 μM for CuONRs and CuONRs@Au_6_NPs. A wider linear curve was observed for CuONRs@Pd_6_NPs and CuONRs@Au_3_Pd_3_NPs within the studied concentration range (0–60 μM) of glucose. The intensity of the absorption band at 652 nm was proportional to the concentration of glucose. Therefore, the amount or concentration of the produced H_2_O_2_ is directly proportional to the concentration of glucose. In the absence of glucose, no absorption signal was observed. The results confirmed that the presence of GOx in solution induces the biocatalytic oxidation of glucose to form gluconolactone. The enzyme was reduced to form GOx(FADH_2_). GOx(FADH_2_) reacts with oxygen to form GOx(FAD) and H_2_O_2_ as a by-product. The linear regression eqn (1)–(4) between the absorbance and varied glucose concentration were:

1 CuONRs: Abs = 0.00376 [glucose] + 0.0663

2 CuONRs@Au_6_Ps: Abs = 0.00372 [glucose] + 0.0708

3 CuONRs@Pd_6_NPs: Abs = 0.00230 [glucose] + 0.102

4 CuONRs@Au_3_Pd_3_NPs: Abs = 0.00305 [glucose] + 0.112

The limit of detection (LoD) and limit of quantification (LoQ) for the CuONRs supported catalysts were investigated. LoD and LoQ values were calculated using *S* = 3*σ* for LoD and *S* = 10*σ* for LoQ, where *S* is a signal and *σ* blank. The LoD and LoQ were calculated using the linear regression eqn (1) for CuONRs, and the LoD was 3.50 μM, and LoQ was 15.80 μM. Eqn (2) was used for LoD and LoQ for CuONRs@Au_6_NPs and were found to be 3.80 μM and 17.10 μM. For CuONRs@Pd_6_NPs, the LoD was 8.87 μM, and LoQ was 39.90 μM, using the linear regression eqn (3). For CuONRs@Au_3_Pd_3_NPs, the linear regression eqn (4) was used, the LoD was 7.34 μM, and the LoQ was found to be 33.10 μM. The selectivity and specificity for glucose detection was attributed to the selectivity of GOx enzyme towards β-d-glucose oxidation. The interference studies were therefore not investigated.

## Conclusions

The preparation of Au_6_NPs-, Pd_6_NPs- and Au_3_Pd_3_NPs-supported on CuONRs was accomplished and confirmed by various methods. The prepared nanomaterials were investigated for their catalytic reduction of H_2_O_2_. The catalytic reduction of H_2_O_2_ by the nanomaterials was affected by different reaction conditions such as pH, reaction time, temperature and varied H_2_O_2_ concentrations. The investigation of the steady-state kinetic parameters showed that the prepared nanomaterials followed the Michaelis–Menten kinetics behaviour. The CuONRs@Pd_6_NPs and CuONRS@Au_6_NPs showed the lowest *K*_m_ values (2.94 mM and 3.11 mM) towards H_2_O_2_, and the values were within range for the HRP enzyme. TMB *K*_m_ values were higher for all our investigated nanomaterials when compared to the HRP enzyme. Nanomaterials demonstrated the effective production of ROS, with various experiments confirming this. The nanomaterials interact with H_2_O_2_ to produce reactive oxygen species (ROS). The prepared nanomaterials were successfully applied for the colorimetric detection of glucose with good linearity, limit of detection (LoD) and limit of quantification (LOQ) were determined.

## Author contributions

S. S.: methodology, investigation, validation, data curation, visualisation, writing – original draft. O. A.: methodology, investigation, validation, data curation, visualisation, writing – review and editing. P. M.: methodology, validation, data curation, visualization, resources, funding acquisition; writing – review & editing, supervision, project administration, corresponding.

## Conflicts of interest

The authors declare no known competing financial interests.

## Supplementary Material

RA-011-D1RA05247K-s001
