# Supplementary material for: Bimetallic gold and palladium nanoparticles supported on copper oxide nanorods for enhanced H2O2 catalytic reduction and sensing
Source: RSC Adv. 2021 Aug 26;11(46):28818–28. doi: 10.1039/d1ra05247k (PMC9038116; doi:10.1039/d1ra05247k)
Supplement: RA-011-D1RA05247K-s001 [file RA-011-D1RA05247K-s001.pdf]

Electronic Supplementary Material (ESI) for RSC Advances.
This journal is © The Royal Society of Chemistry 2021

Electronic Support Information (ESI)

Bimetallic gold and palladium nanoparticles supported on copper oxide nanorods for enhanced H_2O_2 catalytic reduction and sensing

Simbongile Sicwetsha,[a] Omotayo Adeniyi,[a] Philani Mashazi[a,b,*]

[a]Department of Chemistry, Rhodes University, P.O. Box 94, Makhanda, 6140, South Africa.

[b]Institute for Nanotechnology Innovation, Rhodes University, P.O. Box 94, Makhanda, 6140, South Africa.

*Correspondence: p.mashazi@ru.ac.za

Energy dispersive spectroscopy (EDS) was used to investigate the elemental composition of the prepared CuONRs and the gold and palladium supported onto CuONRs. **Figure S1** shows EDS spectra of (a) CuONRs, (b) CuONRs@Au_6NPs, (c) CuONRs@Pd_6NPs, and (d) CuONRs@Au_3Pd_3NPs. The EDS spectrum of CuONRs showed the presence of Cu, O and C. The presence of C emanates from the carbon tape that was used to mount the sample on the stub. The EDS spectra of CuONRs@Au_6NPs, CuONRs@Pd_6NPs and CuONRs@Au_3Pd_3NPs showed additional peaks that are due to Au and Pd nanoparticles. X-ray photoelectron spectroscopy was used for quantitative analysis of the samples.

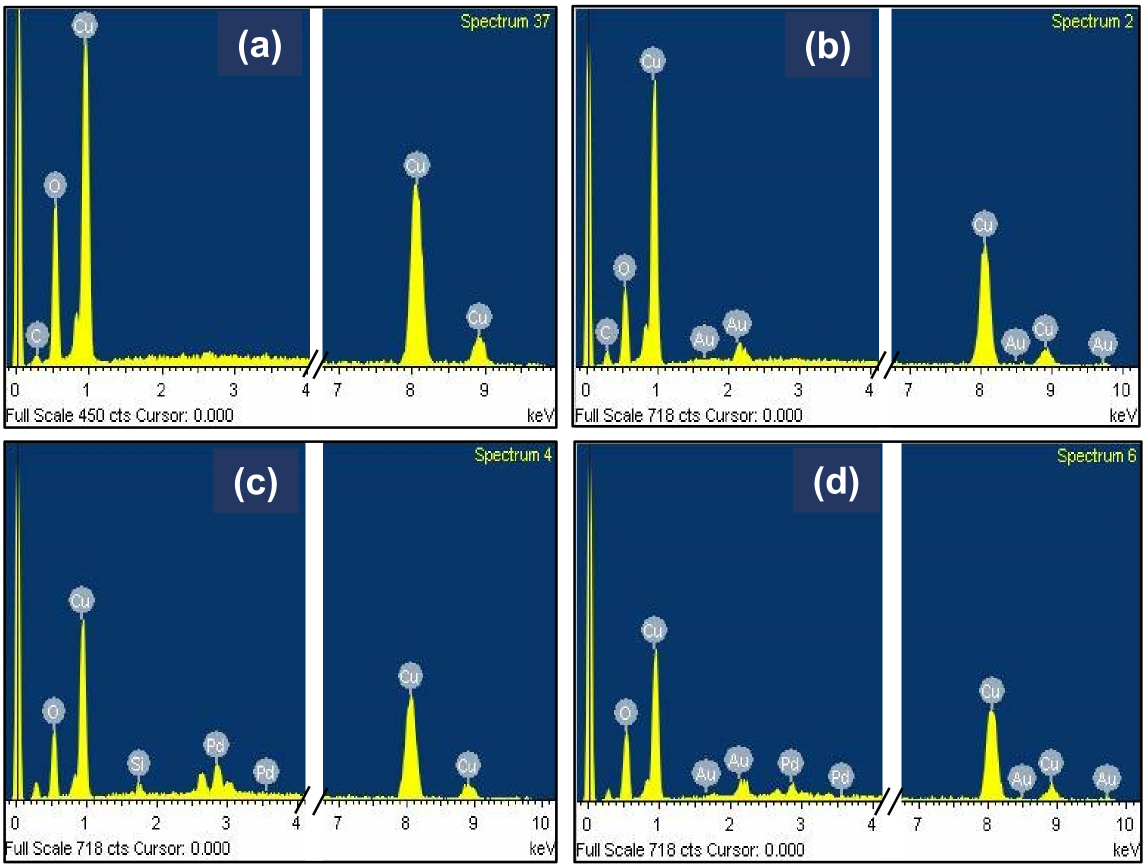

Figure S1: EDS spectra of (a) CuONRs, (b) CuONRs@Au_6NPs, CuONRs@Pd_6NPs, and (d) CuONRs@Au_3Pd_3NPs.

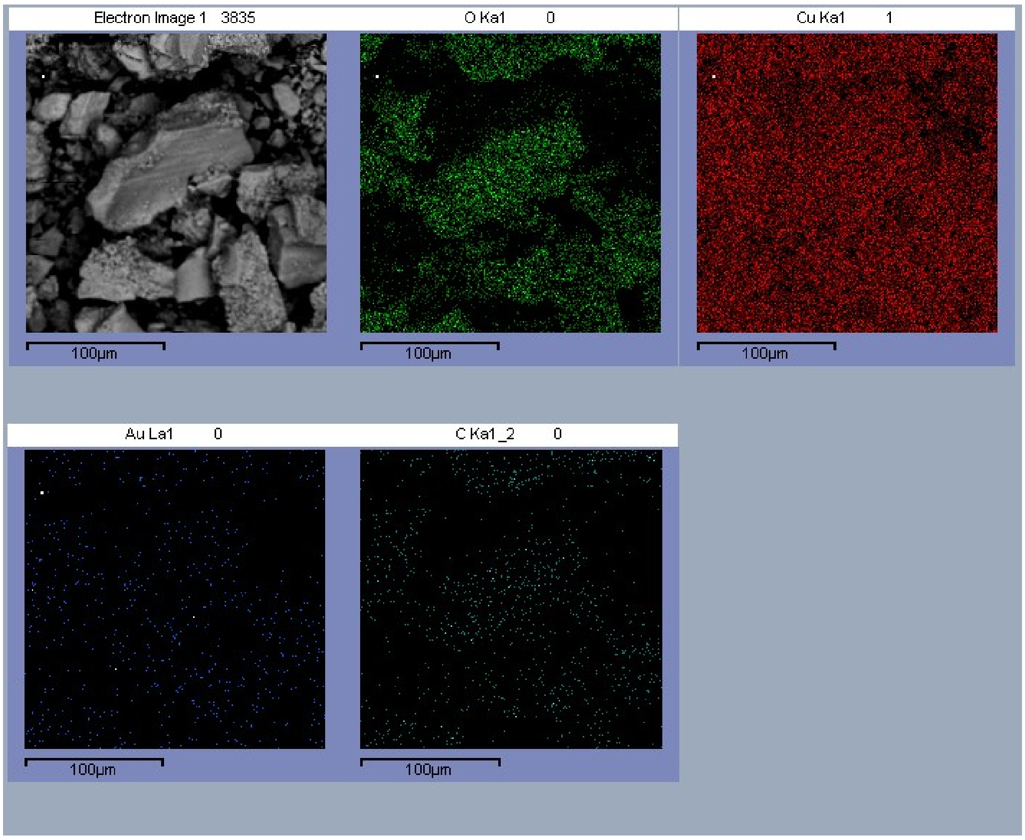

Figure S2: SEM-EDS elemental mapping of CuONRs@Au_6NPs

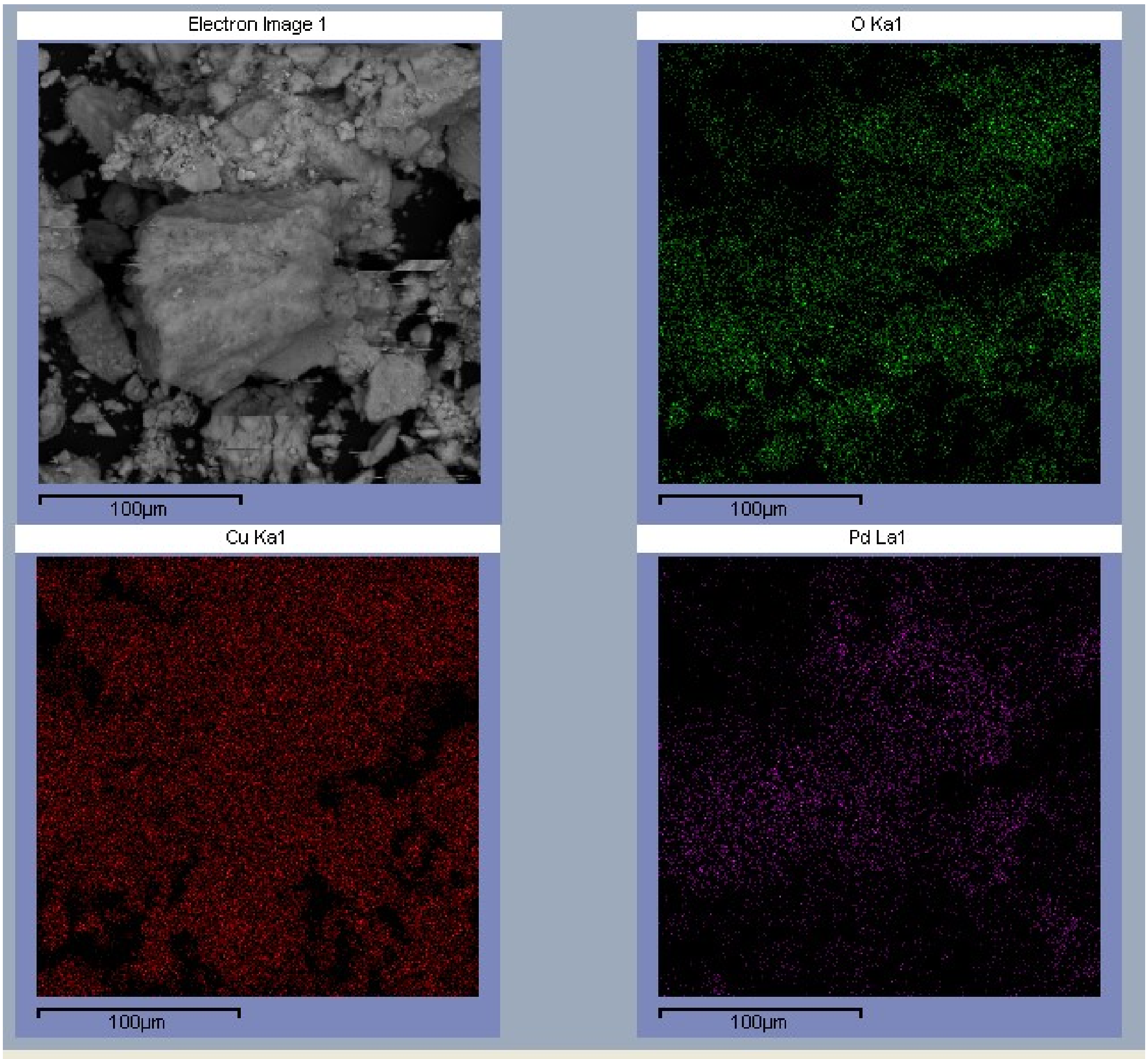

Figure S3: SEM-EDS elemental mapping of CuONRs@Pd_6NPs

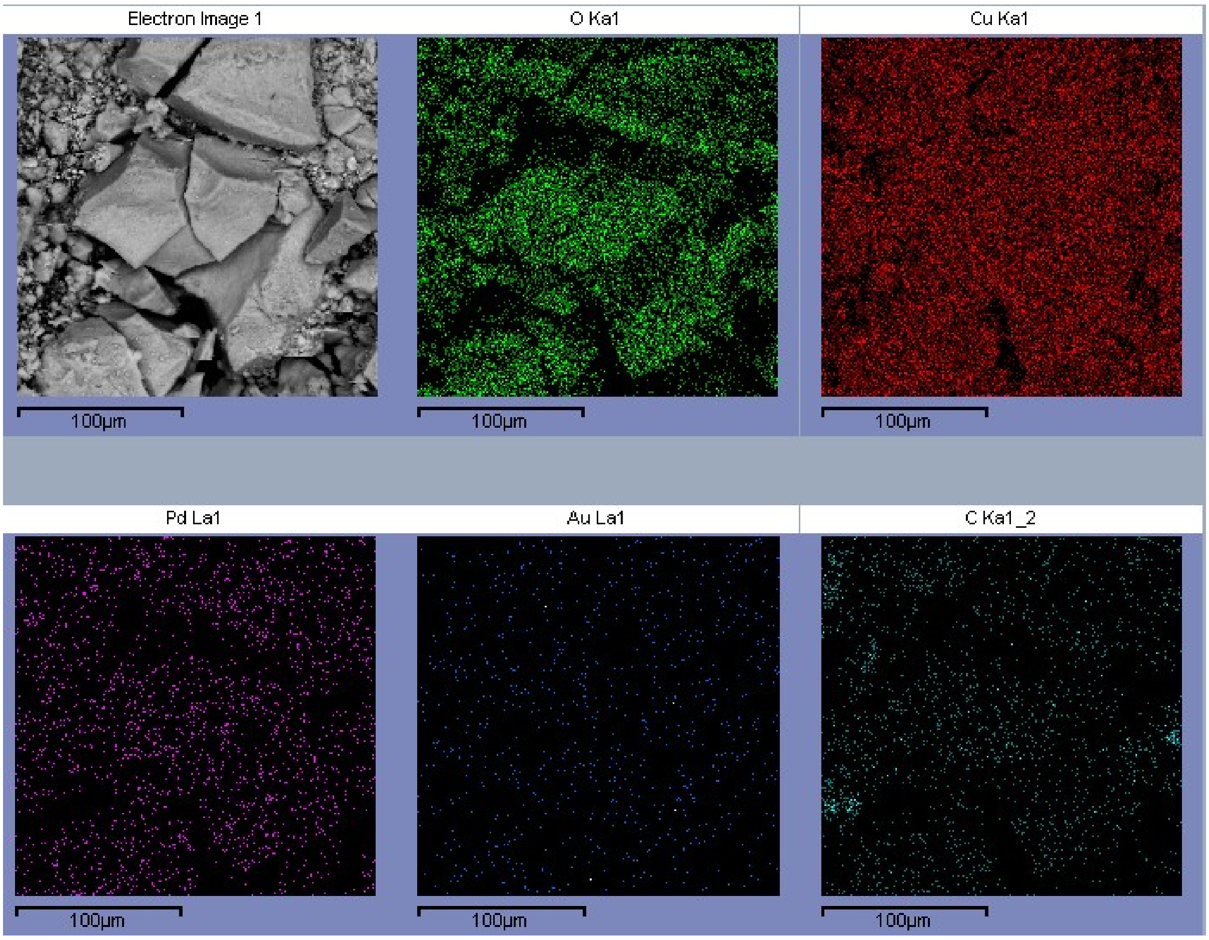

Figure S4: SEM-EDS elemental mapping of CuONRs@Au_3Pd_3NPs.
